# Development and Implementation of a Low-Cost Tracking System after Newborn Hearing Screening in Upper Austria: Lessons Learned from the Perspective of an Early Intervention Provider

**DOI:** 10.3390/children8090743

**Published:** 2021-08-28

**Authors:** Daniel Holzinger, Doris Binder, Daniel Raus, Georg Palmisano, Johannes Fellinger

**Affiliations:** 1 Konventhospital Barmherzige Brüder, Institut für Sinnes-und Sprachneurologie, 4020 Linz, Austria; doris.binder@bblinz.at (D.B.); johannes.fellinger@bblinz.at (J.F.); 2 Research Institute for Developmental Medicine, Johannes Kepler University Linz, 4040 Linz, Austria; 3 Institute of Linguistics, University of Graz, 8010 Graz, Austria; 4 State Health Authority of Upper Austria, 4021 Linz, Austria; daniel.raus@ooe.gv.at (D.R.); georg.palmisano@ooe.gv.at (G.P.); 5 Division of Social Psychiatry, Medical University of Vienna, 1090 Vienna, Austria

**Keywords:** newborn hearing screening, tracking system, implementation study, pediatric hearing loss, enrolment in early intervention

## Abstract

More than one decade after the introduction of newborn hearing screening in Upper Austria, most children were still older than 6 months at enrolment in early intervention. In this study, under the guidance of health authorities, a revised screening and tracking protocol was developed by a network of early intervention providers and representatives of ENT, obstetrics, and pediatrics, including screening professionals and parents of children with hearing loss. Critical process indicators following internationally recommended benchmarks were defined and collected annually by the health authorities. Due to data protection issues, the data collection system was not personalized. Regular network meetings, case-oriented meetings, and screener training sessions were held. As a result, even without additional costs and within the legal constraints related to data protection in Austria, the proportion of children enrolled in early intervention before 6 months of age was significantly increased from 26% to 81% in two representative birth cohorts before and after the introduction of the new protocol, respectively. The coverage for bilateral screening increased from 91.4 to 97.6% of the total number of births.

## 1. Introduction

A recent meta-analysis of permanent hearing loss (HL) of ≥40 dB in the general neonatal population showed a prevalence of 1.33 per thousand for bilateral and 0.78 for unilateral loss [1]. Another meta-analysis to estimate the prevalence of bilateral HL ≥ 26 dB detected by universal newborn hearing screening (NHS) in highly developed countries found a prevalence of 1.1 per 1000 children screened [2]. The World Health Organization (WHO) estimates that at least 34 million children under the age of 15 years have disabling hearing loss [3], and a significant increase between the years 2005 and 2015 has been described [4]. Disabling hearing loss particularly affects speech and language development and consequently cognitive functioning [5,6], academic development [7], behavior [8], mental health [9,10], and quality of life [11], with negative impacts on families and society [12].

Better outcomes in children with HL identified at an earlier age and with timely access to early intervention (EI) are supported by solid evidence. The early provision of hearing aids [13,14] or cochlear implants [14,15,16] has shown strong effects on language learning trajectories. Furthermore, the impact of young age at enrolment in EI on mathematics, literacy [17,18], and socioemotional development [19] has been reported. In a seminal study, Yoshinaga et al. [20] demonstrated significant advantages for language development in children enrolled in EI before the age of 6 months. In addition to the effects of early diagnosis and provision with hearing technology, Holzinger et al. [21] found significant effects for early enrolment in family-centered EI on language development at pre-school ages.

As a consequence, WHO resolutions [22,23] as well as international and national position documents [24,25,26,27] have recommended universal NHS programs as an essential strategy to decrease the impact of congenital HL. Neumann et al. [12] summarized the essential criteria included in these guidelines: (1) coverage of at least 95% of newborns; (2) detection of at least all infants with bilateral HL of ≥35 DB in the better ear; (3) a 4% maximum rate of referrals for audiological testing after failing an NHS; (4) a rate of false positives approaching zero; (5) follow-up with at least 95% of the babies who failed the NHS; (6) a system of direct referrals for screening failures to audiological testing and ENT diagnosis; (7) screening completed before the 1st month of life, audiometric diagnostics completed before 3 months, and beginning EI before 6 months of age, with the Joint Committee of Infant Hearing recently recommending that states that met the 1–3–6-benchmark should even be striving to meet a 1–2–3-month timeline [24]; (8) the inclusion of a data-collection system; and (9) training and supervision of screeners.

However, even though universal NHS has been introduced in many countries (including the majority of high-income countries), many programs still lack comprehensive and mandatory follow-up protocols ensuring high-quality follow-up after failing NHS. Even in advanced programs in North America, the absolute percentages of children receiving timely diagnosis (before 3 months of age) and starting early intervention (before 6 months) are still relatively low. In the “CDC’s Hearing Screening and Follow-up Survey 2006–2016”, Krishnaveni et al. [28] reported that 36.6% of infants received diagnostic testing before 3 months of life and that 47.2% were enrolled in EI before 6 months. In a systematic review involving 46 studies, the total rate of failure to follow-up was 20–21% without adherence to time benchmarks [29]. For Bavaria, Brockow et al. [30] reported that the screening center had to intervene in 37.9% of the cases to ensure follow-up for infants who had failed NHS. Globally, educational disparities and lack of knowledge among parents, distance, and work constraints were reported as the most frequent factors leading to failure to follow-up [29]. Other factors delaying or preventing follow-up that cannot be directly influenced by a standardized tracking protocol are family-related factors, such as parental denial of atypical screening results and reluctance to enroll in EI, or medical conditions that can complicate the diagnostic assessment.

Despite the evident need for comprehensive NHS programs including tracking systems, only a few studies describe the actual implementation process and outcomes of specific programs. This study aims to report the development and implementation of a low-cost tracking system to follow infants from NHS failure, through ENT confirmatory diagnosis, to enrolment in a family-centered EI in Upper Austria within the legal constraints of data protection in Austria. The effects of the newly established screening and tracking protocol on central process indicators including age at the start of EI are reported.

## 2. Materials and Methods

### 2.1. Demography

Upper Austria is one of nine Austrian states and has a population of 1.49 million inhabitants and a current birth rate of about 15,000 per year. The majority of infants are born in 1 of 14 maternity hospitals. Only 1.6% of all babies born in Austria are delivered at home [31]. The Upper Austrian health system provides six hospital-based ENT departments; four of them offer specialized pediatric services. Specialized EI for children with HL and their families is provided exclusively by the Family-centered Linz Intervention Program (FLIP), which follows internationally established best practice guidelines for family-centered EI in children who are deaf or hard of hearing [32]. Access to medical care and EI is provided free of charge.

Following the recommendation of a position paper by the Austrian ENT Society (Millstatt Concept), universal NHS was introduced in Upper Austria in 1995. The position paper recommended the administration of NHS as a two-stage OAE (otoacoustic emissions) screening of at least one ear and documentation of the results in the so-called “mother–child passport” (Mutter–Kind Pass). In the case of a screening failure, the children should be referred for ENT audiological diagnostics. The Millstatt Concept did not contain any details about tracking follow-up after NHS failure, recommendations for screening-personnel and screener training, the responsibilities of screening programs as a whole, or the necessity for a network of professionals and stakeholders. In a study evaluating NHS in Austria 10 years after its first implementation using retrospective chart reviews of 321 children with hearing loss [33] registered at ENT departments or institutions for children with HL over a period of 2 decades, 35% of the children screened (vs. 2% of those unscreened) had completed medical diagnosis of HL before the age of 3 months and 69% of the children had started intervention at the age of 6 months compared with 4% of those who had not been screened. Although almost all Austrian hospitals with maternity wards have introduced NHS, it still is not mandatory in Austria.

### 2.2. Development of a Screening and Tracking Protocol

Documentation of the age at enrolment in early intervention (FLIP) over the years demonstrated that, despite the implementation of universal NHS and a high coverage rate in Upper Austria, the majority of children were older than the internationally recommended benchmark at enrolment in EI. In 2012, 52% of the children enrolled in FLIP were older than 6 months of age. As a consequence, the EI program leaders contacted the health authorities of Upper Austria in 2012 with the aim of establishing a more effective screening program under governmental guidance of the state of Upper Austria, including a tracking system following NHS failure, through medical diagnosis, to enrolment in EI.

As a first step, a survey to collect information about the current state of the NHS procedures was sent out to all medical directors of maternity wards, ENT, and pediatric departments. In the year 2012, the maternity wards reported unilateral screenings in 97% of the neonates and bilateral screenings in 91.4% of them. Four out of fourteen maternity wards used AABR in addition to OAE screening, whereas the majority exclusively used screenings by OAE. Screenings were performed by a total of 155 professionals, mainly nurses and secondarily speech-language therapists. Four of the maternity wards could not report exact data on the number of children who left the hospital as having failed the screening. Of those for which data could be provided, the rate of infants who failed the screening that left the maternity ward was 3.49%. All of the maternity wards reported that all parents had been informed about NHS. In addition to an insufficient rate of bilateral screenings, insufficient documentation (e.g., on screening failures or ENT follow-ups before 3 months of age), and a high rate of infants with delayed enrolment in EI, communication within the network demonstrated a number of shortcomings in the current practice: responsibility for the administration and central oversight of NHS was not regulated. Furthermore, the maximum number of screening repetitions and the ENT departments to which infants who failed the NHS should be referred were not specified. Parents of babies who left the maternity ward with an abnormal NHS result were only advised to make an ENT appointment while appointments were not organized for them. Overall, neither a tracking system that includes specific procedures in case of non-appearances for ENT diagnostics, nor a guideline for referral to EI after medical confirmation of HL were installed.

Based on the available information and international NHS guidelines, the standard operating procedures of a new NHS program including tracking of infants who failed NHS were specified in several working meetings with health authorities and EI providers. Due to data protection issues in Austria, the introduction of a personalized documentation system was not possible. Moreover, the Austrian federal government had decided not to include NHS documentation in a national birth register. As NHS is still not mandatory in Austria and hospitals are not specifically paid for NHS, the new program had to be planned in a way that saves resources and time. In 2014, the proposal of a new NHS program was presented to the representatives of all maternity wards and pediatric departments and to those of ENT units and was slightly adapted. The final condensed guideline for newborn hearing screening and tracking is presented in Table 1.

A minimal data collection system to monitor process indicators was developed. The information to be collected from maternity wards included the total number of births, the number of children screened once and the number that completed screening, the screening methods of the first and second screening, the number of infants for whom an appointment for ENT diagnostics was organized, and the specific ENT department(s) eligible for the assessment of infants. The ENT departments were to report the number of appointments for medical-audiological diagnosis of hearing loss that had been organized by the maternity wards. In addition, the proportion of families who kept their first appointments, the number of additional invitations (after not having kept the initial appointment), the final number of appointments, the number of children with confirmed hearing loss (unilateral or bilateral), the number of children with completed ENT diagnosis before their 3rd month of life, and the number of children referred for EI were collected. Finally, the EI providers were asked to provide the number of cases referred by the ENT departments, the number of families contacted, the number of children enrolled in EI, the proportion of those contacted within 48 h after referral, and the proportion of those enrolled in EI within two weeks after first contact.

### 2.3. Implementation of the New NHS Program

In 2015, the implementation started under the guidance of the Upper Austrian state health authority. Each of the maternity wards and ENT departments was asked to appoint at least one member of staff responsible for NHS. Since 2015, the minimal data set of process indicators described above has been annually collected. Network meetings organized by the health authority involving medical representatives (obstetrics, ENT, and pediatrics) and screeners, EI providers, and parent representatives as well as the governmental mandates themselves were held in 2015, 2016, and 2017. Information about the process indicators was shared and discussed, and experiences with the new NHS program were exchanged. In addition, parents of children who are deaf or hard of hearing shared their experiences with NHS. The meetings were also used for information transfer regarding different aspects (e.g., etiologies and outcomes) of pediatric HL.

In 2016/2017, a curriculum for screener training was developed with support from UK NHS specialists, and two half-day screener trainings with about 40 participants each were held in the following years. In addition to exchanging and consulting about the practical implementations of screening and documentation, the screeners were specifically trained in communicating about NHS and the screening results with the parents. The involvement of parents in the screener trainings was found by the participating screeners to be very helpful.

In addition to a regular interdisciplinary case discussion of early intervention providers with one of the ENT departments specialized in pediatrics for over almost 2 decades, meetings with another ENT unit were organized more regularly and regular meetings with a third ENT unit were initiated. In addition, case-oriented exchanges with the other otorhinolaryngological units with smaller sizes were intensified.

### 2.4. Statistical Analysis

The descriptive data on most process indicators were available exclusively for the time period after the introduction of the new NHS program. However, comparative data on the age at enrolment in EI are available for two birth cohorts (2011 and 2017) before and after implementation of the new protocol. For comparisons between birth cohorts, χ²-Tests were conducted for categorial variables, and due to the small sample size and non-normal variables, Mann–Whitney U Tests were conducted for metric variables (age).

## 3. Results

A high rate of screenings after birth and a young age at enrolment in EI can be regarded as the central process indicators of an effective NHS program. When the first survey was conducted in 2012, the percentage of neonates screened unilaterally was 97% and that for bilateral screenings was 91.4%. In 2017, 97.6% of all newborns in Upper Austria were screened bilaterally. The coverage for bilateral screening in 2017 was statistically significantly higher than in 2011 (χ²(1) = 540.4, *p* < 0.001). This figure exceeds the minimal 95% coverage recommended by international guidelines. On average, 3.59% of the screened infants left the hospital with a positive screening result. This rate of referrals for audiological testing after failing NHS, defined as an essential criterion by the position paper, is below the maximum rate of 4%. In 2 of the 14 maternity wards, the percentage exceeded the 4% benchmark. Our data indicate that all children (*n* = 530) who had been referred for ENT diagnostics finally arrived in one of the designated departments. Only 51 of the families (9.6%) had to be reminded of their appointment. From a total of 22 children with significant sensorineural hearing loss, 21 were referred to EI. In addition, ENT departments reported the identification of eight children with permanent conductive hearing loss.

Enrolment in early intervention involves fitting with hearing technology and consequently access to spoken language. Support for the families in their everyday use of hearing aids or cochlear implants, in coping with the diagnosis, and in the apaptation of their communication and language to their child’s current needs is as important as provision with hearing technology. Therefore, a young age at the start of early intervention is critical for the optimization of outcomes.

**Figure 1:**
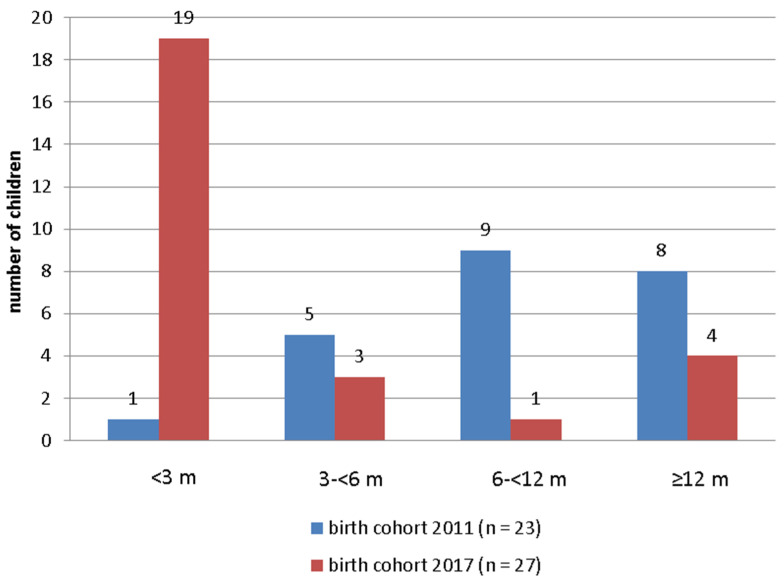
Comparison of age at enrolment in EI of birth cohorts 2011 and 2017.

Figure 1 presents the comparative data on age at enrolment in EI for the birth cohorts 2011 and 2017. To include as many children as possible, particularly latecomers, the enrolment data of four consecutive years (2011–2014 and 2017–2020) are included. Within periods of 4 years including the year of birth, 23 children born in 2011 (1.7 per thousand births) and 27 children born in 2017 (1.75 per thousand births) were admitted to EI.

The comparison of age at enrolment in EI of the birth cohorts 2011 and 2017 (Figure 1) demonstrates a significant increase in the proportion of children younger than 6 months of age in the younger cohort after the introduction of the new NHS and tracking protocol (Mann–Whitney U = 94.0, *p* < 0.001). The percentage of children below the benchmark of 6 months increased from 26% (birth cohort 2011) to 81% (birth cohort 2017). The mean age at enrolment was reduced from 11.5 to 4.8 months (medians 7.8 to 2.0). The high number of children younger than 3 months of age at enrolment in EI (*n* = 19; 70%) compared with only one child in the 2011 cohort (4.3%) is remarkable.

## 4. Discussion

The implementation of a jointly developed quasi-mandatory standard operating procedure for NHS and tracking of screening failures under the guidance of health authorities helped to significantly improve the effectiveness of an NHS program that had been introduced two decades before without additional costs and with the use of minimal documentation. The coverage of bilateral screenings increased from 91.4 to 97.6% of annual births. The mean age at enrolment in EI was reduced from 11.5 months (median 7.8) in the birth cohort 2011 to 4.8 months (median 2.0) in the birth cohort 2017. After the introduction of the new protocol, EI was initiated in 81% of the children before the age of 6 months compared with the 26% previously. Compared with international data for high-income countries, the young age of enrolment in EI in Upper Austria can be regarded as a significant achievement. As a comparison, Krishnavenii [28] reported a rate of 47.2% for the year 2016 for North America. The available data on age at the start of EI studies after NHS usually refer to the age at hearing-aid fitting rather than the age at enrolment in family-centered intervention programs. For a comparable sample of Australian children undergoing EI (after exclusion of those with a device fitting later than 3 years), a mean age of 9.1 months (median 5.1) was reported [14]. For the highly developed NHS program in England, a comprehensive study including about 4.6 million children born between 2004 and 2013 [34] found a median age at hearing-aid fitting of 82 days.

A number of key factors most likely account for the successful alterations that were achieved and for the lessons we learned during the process.

Establishing a multi-professional network of professionals including staff of maternity wards, pediatric units, ENT departments with pediatric specialization, and early EI providers under the guidance of health authorities is mandatory for the development and practical implementation of a successful NHS program. In addition to multi-professional network meetings organized by health authorities to discuss quality issues within the NHS program, we found that regular case discussions between ENT representatives and EI providers are essential for improvements in the transition between medical diagnostics and EI. Screener workshops for an exchange of experiences and for training purposes are another critical component of the NHS network. In addition, the involvement of parents of children with hearing loss in the network helps to adjust the procedures to parental needs.

Continuous active guidance of the health authorities is essential for initiation of the multi-professional and multi-system process as well as for quality assurance, which is mainly achieved by regular collection of process indicators. Even though data protection issues in the state of Upper Austria and the governmental decision to not include NHS documentation in a national birth register prevented the introduction of a personalized documentation system, minimal documentation of the process indicators helped to improve the outcomes. The process indicators collected annually by health authorities should refer to the essential criteria defined by international guidelines, in particular the number of infants at ages within the established benchmarks for the completion of screening, diagnostics, and initiation of EI. As early intervention is often understood as the fitting of hearing technology, we emphasize, for the reasons mentioned above, the importance to include the age at enrolment in a family-centered EI program (ideally over a period of several years after birth) among the critical process indicators. Even though the effectiveness of NHS was significantly improved by using a minimal dataset, a personalized documentation system including records from birth, of the NHS, of the medical-audiological diagnostics, and of enrolment in EI would be preferable with regard to the reliability of the data. Furthermore, more documentations allows for investigations of the causes of delays in intervention on an individual basis.

The joint adaptation of internationally suggested models of documentation to the national or regional resources and legal conditions helped to make documentation feasible and to support cooperation among the screeners, ENT departments, and EI providers.

The Austrian federal system also employs different regulations and practices of health care in the nine states. Therefore, we decided to establish a model limited to a single state but that is highly comprehensive concerning the involvement of representatives active in all stages from NHS to EI. Effective small case models lend themselves for later expansions to larger regions. The results of the new screening and tracking protocol were regularly reported at the annual conference of the Austrian ENT society and stimulated the development of a new guideline on screening, assessment, and care [35] of congenital HL.

Finally, we learned from the Austrian experience that an NHS program is only rendered effective by the existence of and adherence to a tracking system that guarantees timely follow-up from failing NHS to enrolment in EI. The limitation of the number of screening repetitions (not more than two), the maternal wards’ direct arrangement of appointments with specified ENT departments, and the active and timely invitation of families for enrolment in EI by the EI providers can be considered key factors. A number of persistent challenges need to be considered for successful continuation of the program. About half of the professionals administering the screening did not attend the training program so far. All of the ENT professionals involved in the diagnosis of hearing loss need to be reminded of the impact of even mild or unilateral hearing loss and the necessity to refer *all* children with hearing loss to EI. The relevance of making appointments for ENT diagnosis by the maternity wards rather than simply referring them needs to be continuously emphasized.

The implementation of a new screening and tracking protocol in the state of Upper Austria that includes data from the only specialized EI program available can be regarded as a strength of this study. The proportion of children with identified hearing loss in the birth cohorts 2011 and 2017 who were admitted to EI supports the epidemiological characteristic of this study. However, the identification of HL in children who failed NHS even after a period of three years after the year of birth cannot be excluded. In addition, the reliability of data reported by the maternity wards and ENT departments is not completely verifiable due to the non-personalized data collection system.

## 5. Conclusions

After NHS was introduced in Austria in 1995, the introduction of a screening and tracking protocol in the state of Upper Austria under the active guidance of health authorities, the establishment of professional networks, and the annual collection of critical process indicators helped to significantly lower the age at enrolment in early interventions without additional costs and within the national legal constraints.

## Figures and Tables

**Table 1:** NHS and tracking protocol for Upper Austria.

Time Sequence	Actions
Before delivery	First time parents are informed about NHS at their first meeting at the maternity ward
2nd to 3rd days of life	Informing parents about NHS. Obtaining parental consent to perform NHS, to document it alongside birth information, and to transfer contact information to the ENT department in case of a screening failure. Performance of NHS: 1. Oto acoustic emissions (OAE) 2. In the case of abnormal results from the 1st screening, the screening (OAE or AABR) is repeated before discharge from the maternity ward. In the case of abnormal results from the 2nd screening, a referral is made to a specialized ENT department and a timely appointment is arranged. Additionally, parents may contact a known ENT doctor in private practice.
Within the first 4 weeks of life (only after outpatient or home birth)	In the case of outpatient or home birth, parents are advised to arrange for NHS at a maternity ward, ENT department, or private ENT practice.
Documentation of the results in the mother–child passport and documentation system of the maternity ward	
Around the 3rd week of life	In default of appearance for ENT diagnostics, parents are reminded of the appointment by telephone by the ENT department.
From the 4th week of life	A pediatrician/general practitioner checks the NHS results (documented in the mother–child passport) and refers the child to ENT diagnostics, including arranging the new appointment
By the 3rd month of life	ENT diagnostics: Brainstem audiometry (preferably frequency specific) during natural sleep
In case of confirmed sensorineural HL and/or permanent conductive hearing loss	Referral to a specialized early intervention program after having obtained parental consent; transfer of the contact information (name and telephone number) to the EI program; after enrolment in EI, timely referral to incremental care
Within 48 h after first information	Contact from the EI program via telephone with parents to arrange a first meeting (within 10 days)

## Data Availability

Restrictions apply to the availability of these data. Data were collected from the state health authority Upper Austria and has not been made publicly available.
